# Effects of selective serotonin reuptake inhibitor treatment on plasma oxytocin and cortisol in major depressive disorder

**DOI:** 10.1186/1471-244X-13-124

**Published:** 2013-04-29

**Authors:** Charlotte Keating, Tye Dawood, David A Barton, Gavin W Lambert, Alan J Tilbrook

**Affiliations:** 1 Monash Alfred Psychiatry Research Centre, Central Clinical School, Monash University and The Alfred, Melbourne, Australia; 2 Brain and Psychological Sciences Research Centre, Swinburne University of Technology, Hawthorn, Australia; 3 Human Neurotransmitters Laboratory, Baker IDI Heart & Diabetes Institute, Melbourne, Australia; 4 Mental Health Program, Southern Health, Melbourne, Australia; 5 Faculty of Medicine, Nursing & Health Sciences, Monash University, Melbourne, Australia; 6 South Australian Research and Development Institute, The University of Adelaide, Adelaide, Australia

**Keywords:** Major depressive disorder, Oxytocin, Cortisol, Hypothalamopuitary adrenal axis, SSRI

## Abstract

**Background:**

Oxytocin is known for its capacity to facilitate social bonding, reduce anxiety and for its actions on the stress hypothalamopituitary adrenal (HPA) axis. Since oxytocin can physiologically suppress activity of the HPA axis, clinical applications of this neuropeptide have been proposed in conditions where the function of the HPA axis is dysregulated. One such condition is major depressive disorder (MDD). Dysregulation of the HPA system is the most prominent endocrine change seen with MDD, and normalizing the HPA axis is one of the major targets of recent treatments. The potential clinical application of oxytocin in MDD requires improved understanding of its relationship to the symptoms and underlying pathophysiology of MDD. Previous research has investigated potential correlations between oxytocin and symptoms of MDD, including a link between oxytocin and treatment related symptom reduction. The outcomes of studies investigating whether antidepressive treatment (pharmacological and non-pharmacological) influences oxytocin concentrations in MDD, have produced conflicting outcomes. These outcomes suggest the need for an investigation of the influence of a single treatment class on oxytocin concentrations, to determine whether there is a relationship between oxytocin, the HPA axis (e.g., oxytocin and cortisol) and MDD. Our objective was to measure oxytocin and cortisol in patients with MDD before and following treatment with selective serotonin reuptake inhibitors, SSRI.

**Method:**

We sampled blood from arterial plasma. Patients with MDD were studied at the same time twice; pre- and post- 12 weeks treatment, in an unblinded sequential design (clinicaltrials.govNCT00168493).

**Results:**

Results did not reveal differences in oxytocin or cortisol concentrations before relative to following SSRI treatment, and there were no significant relationships between oxytocin and cortisol, or these two physiological variables and psychological symptom scores, before or after treatment.

**Conclusions:**

These outcomes demonstrate that symptoms of MDD were reduced following effective treatment with an SSRI, and further, stress physiology was unlikely to be a key factor in this outcome. Further research is required to discriminate potential differences in underlying stress physiology for individuals with MDD who respond to antidepressant treatment, relative to those who experience treatment resistance.

## Background

Oxytocin is a neuropeptide that is synthesised in the supraoptic (SON) and paraventricular (PVN) nuclei of the hypothalamus. It is released into the brain via distributed oxytocinergic pathways and oxytocin receptors are located in various socially relevant and stress-sensitive brain regions [1]. This neuropeptide can facilitate maternal-infant attachment and pair-bond formation in a variety of mammals [2-4], as well as decrease anxiety and stress [5] when centrally administered [6]. Oxytocin is also released in response to acute psychogenic stressors in mammals [6,7].

Clinically, oxytocin has been shown to facilitate improved social communication behaviour in humans (e.g., eye contact, nonverbal positive behaviour and self-disclosure). In an investigation involving healthy controls, oxytocin compared with placebo reduced couple conflict (improved social communication) and reduced cortisol concentrations [8]. Multiple investigations have also demonstrated the therapeutic application of oxytocin for anxiety symptoms. For example, oxytocin has been shown to enhance the buffering effect of social support on stress responsiveness [9]. Oxytocin or placebo was administered to healthy controls prior to exposure to the Trier Social Stress Test (TSST) in the presence of participants’ best friend or no social support [9]. Compared to placebo, those administered oxytocin in the context of social support experienced increased calmness and decreased anxiety during the TSST, and showed the lowest salivary free cortisol concentrations [9]. In addition, in patients with social anxiety disorder, oxytocin compared to placebo was associated with improved positive evaluations of appearance and speech performance as exposure treatment sessions progressed [10]. These findings suggest that oxytocin can reduce symptoms of anxiety which is likely, due in part, to attenuating the activity of the HPA axis [8]. Although oxytocin can reduce anxiety and attenuate activity of the hypothalamo-pituitary adrenal (HPA) axis, [5,11] the precise mechanisms by which this occurs are not yet well defined.

These findings suggest the possibility that oxytocin may be of clinical relevance to disorders associated with psychosocial impairment, anxiety and dysregulation of the HPA axis. Individuals with major depressive disorder (MDD) often experience difficulties in interpersonal relationships, and social isolation. Furthermore, up to 60% of patients with MDD experience elevated activity of the HPA-axis [12]. Dysregulation of the HPA system is reportedly the most prominent endocrine change seen in MDD and normalizing the HPA axis is one target of recent treatment developments [13-15].

Several lines of evidence support that the oxytocin system is altered in MDD. For example, a small sample of patients with MDD (n = 3) showed a 23% increase in oxytocin neurons in the PON of the hypothalamus compared with matched healthy controls [16]. It was concluded that this increase may be linked to increased HPA axis activity, commonly reported in patients with MDD [16]. Increased staining for oxytocin neurons has since been demonstrated in other research [17]. Potential differences in oxytocin, cortisol and arginine vasopressin concentrations in patients with MDD (n = 11) compared with matched healthy controls (n = 19) have also been investigated [18]. Results revealed increased oxytocin concentrations in individuals with MDD compared to healthy controls, whereas no differences were observed in cortisol or arginine vasopressin concentrations [18]. Elevated oxytocin concentrations found in MDD is also consistent with evidence that oxytocin mRNA levels are increased in depressed patients (e.g., [16,18]).

The potential clinical relevance of oxytocin to MDD has also been investigated. Plasma oxytocin concentrations have been linked to the temperament dimension of reward dependence (according to Temperament and Character Inventory, TCI) in patients with MDD [19]. In other research, although an association between performance on neuropsychological testing and elevated basal cortisol concentrations was demonstrated in MDD, no association was found between performance and plasma concentrations of oxytocin [20]. Significant correlations have also been shown between oxytocin concentrations and depressive symptoms in several studies involving individuals with a diagnosis of: obsessive compulsive disorder [21], fibromyalgia [22] and MDD [23,24].

A relationship between oxytocin and treatment outcomes has also been investigated in MDD. In one study involving patients with MDD or bipolar disorder, oxytocin concentrations were not influenced by treatment (involving either an SSRI, tricyclic antidepressant or electroconvulsive therapy) [25]. These findings are in agreement with previous results demonstrating no difference in cerebrospinal fluid oxytocin concentrations in medicated, symptomatic individuals with MDD compared to healthy controls [26]. Patients included in the study were taking heterogeneous treatments, however, (10 patients were treated with a tricyclic antidepressant, two with a monoamine oxidase inhibitor, two with lithium carbonate and one with an antipsychotic [26]) which may have influenced the outcomes.

Preclinical evidence indicates that oxytocin can influence activity of the serotonin system, which may relate to the antidepressant effects of SSRIs [27]. In a recent study in mice, widespread expression of oxytocin receptor containing cells were shown to be co-localised with tryptophan hydroxylase-positive neurones in the raphe nuclei [28]. Furthermore, intra-raphe infusion of oxytocin increased serotonin release within the median raphe, a response that was inhibited by serotonin 2A/2C receptor antagonists. It was speculated thus that the increased serotonergic activity induced by oxytocin may underlie its anxiolytic effects [28]. Given the use and effectiveness of SSRI’s in MDD, these findings may also relate to their antidepressant-like effects [27].

Further, evidence supports that the serotonin and oxytocin systems may interact in the hypothalamus [29]. In macaques, neuroanatomic evidence shows that the distribution of serotonin transporter fibres follows the distribution of oxytocin cells in the hypothalamus in the parvicellular, magnocellular, dorsal, and posterior subdivisions of the paraventricular nuclei. In the supraoptic nuclei, serotonin transporter fibers and oxytocin cells overlap in the ventromedial subdivision and in the ‘capsular’ part of the dorsolateral supraoptic nuclei [29]. Again, it has been concluded that these findings provide neuroanatomical support that SSRIs’ therapeutic effects may be mediated in part, through components of the oxytocin system [29].

Although a number of pharmacological agents are available to treat depression, at least 30-40% of patients do not respond to these [30,31]. Therefore, there is a major emphasis in psychiatry to uncover the underlying aetiology of mood disorders, to be able to develop more effective treatments [27]. In this respect, oxytocin may be of therapeutic benefit in patients with MDD. The primary aim of this study was to gain further understanding of the role of oxytocin in the pathophysiology of MDD. Using an open label treatment design we examined whether there is a relationship between oxytocin concentrations and symptoms of MDD and their resolution following (SSRI Treatment. In a secondary aim, we intended to investigate whether there is a clinically relevant relationship between oxytocin and cortisol in relation to symptoms of MDD and their resolution.

## Methods

### Experimental design

In this study we extended an open label investigation of SSRI treatment in patients with MDD [32]. Arterial sampling was performed twice, first while patients were untreated during a current depressive episode and then following 12 weeks treatment with an SSRI. For each patient, the samples were collected at the same time of day. All patients involved in this study were treatment responders. For all participants, 12 hours preceding study visits, caffeine, alcohol and tobacco smoking were prohibited. The research protocol was approved by the Alfred Hospital ethics review committee. Written informed consent was obtained from each subject prior to the study.

The study included 16 participants with MDD (9 females, 7 males) ranging in age from 22 yrs to 71 yrs (mean ± SEM, 43.5 ± 3.3 years) who met DSM-IV criteria and International Statistical Classification of Diseases, 10th Revision (previously described in [32]). Patients were either newly diagnosed or currently untreated after a relapse and had not been receiving antidepressants or benzodiazepines for at least 4 weeks prior to the study (5 weeks if they had been receiving fluoxetine hydrochloride) (see Barton et al., 2008). Two females were taking hormone-based contraceptives, two were postmenopausal, and information was not available for the remaining four individuals. Stage of menstrual cycle was not available. Patients were initially screened via telephone and interviewed by a psychiatrist (D.A.B.) using a structured clinical interview (Mini International Neuropsychiatric Interview, MINI). The 17-item Hamilton Depression Scale (HAM-D), Spielberger’s State and Trait Anxiety Inventory (STAI), and Beck Depression Inventory (BDI) were used to assess severity and monitor response to treatment. Patients were eligible for inclusion if they fulfilled criteria for MDD on the MINI, had HAM-D and BDI scores of 18 or higher and were assessed as having MDD as the primary illness at psychiatric interview. Patient selection aimed to minimize psychiatric co-morbidity. The Anxiety Disorders Interview Schedule for DSM-IV was used to discriminate between anxiety disorders and for determining the primary and secondary diagnosis based on the participant’s responses and severity scores on measures of symptoms. Clinical significance was defined as a score of 4 or higher on the 8-point Likert-type scale, where 2 is mild, 4 is moderate, 6 is severe, and 8 is very severe. Patients presenting with co-morbid panic or anxiety were included if the primary diagnosis was depression and any panic or anxiety was secondary to their depression. Participants were excluded if they had coexisting heart disease, diabetes, medicated hypertension, alcohol or drug abuse or dependence, or infectious disease; had a co-morbid psychotic disorder, eating disorder, mental retardation, personality disorder, or epilepsy; or had a current high suicide risk. Patients having previously failed to respond to SSRI treatment at the maximum tolerated dose for at least 4 weeks were excluded from the study.

Within 10 days of confirming diagnosis of MDD, initial research studies were performed. Patients then commenced treatment with an SSRI which was determined on clinical grounds in consult with the participant (12 received citalopram hydrochloride, 2 received sertraline hydrochloride, 2 received fluoxetine). No structured psychotherapy was provided to the patients in the context of the research study, or external to it. Repeat research studies with arterial blood sampling was performed after approximately 12 weeks of therapy. Patients were examined weekly for the purposes of the study or more frequently if required on clinical grounds (as previously described [32]). Significant clinical improvement was defined as a decrease of more than 50% in HAM-D scores and remission as a HAM-D score lower than 8.

### Oxytocin cortisol radioimmunoassays

Arterial concentrations of oxytocin were measured before and after SSRI treatment by radioimmunoassay using Phoenix Pharmaceuticals Oxytocin RIA Kit (Belmont, California, USA) [33]. There was 100% cross-reactivity of the oxytocin antibody with oxytocin and no cross-reactivity with arginine vasopressin. All samples were measured in a single assay. Cortisol concentrations were measured in plasma collected before and after SSRI treatment using an extracted radioimmunoassay [34] using hydrocortisone (H-4001, Sigma Chemical Company, St Louis, MO, USA) as standard. The assay utilized [^3^H]-cortisol (Amersham Pharmacia Biotech UK, Buckinghamshire HP, England) as tracer and a dichloromethane extraction procedure with a mean (± SEM) recovery of 93.2 ± 2.8%. All samples were measured in a single assay, which had a sensitivity of 0.44 ng/ml.

### Statistical analyses

We tested the normality of the distribution of oxytocin data by conducting a Kolmogorov-Smirnov statistic. The primary variable of interest, oxytocin, was not normally distributed. Therefore, we conducted nonparametric statistics: Mann–Whitney test, Wilcoxon sign ranked test, and Spearmann’s rho correlations.

Wilcoxon ranks test was used to assess differences in oxytocin and cortisol concentrations before (untreated) compared to following (treated) SSRI therapy. Pairs of dependent variables were: arterial oxytocin concentrations (A1 and A2), cortisol concentrations (visit 1 and visit 2), as well as psychopathology scores, HAMD (visit 1 and visit 2), BDI-I (visit 1 and visit 2), STAI state (visit 1 and visit 2) and STAI trait scores (visit 1 and visit 2).

## Results

### Symptoms of MDD and anxiety

**Figure 1:**
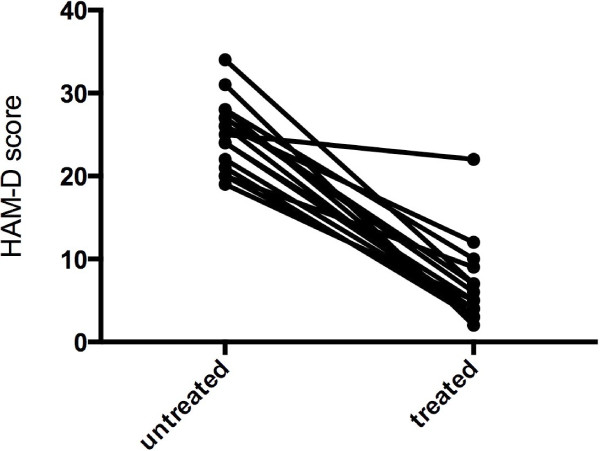
HAM-D scores in patients with MDD, untreated and following SSRI treatment.

Patients with MDD were moderately depressed with a mean (±SEM) HAM-D score of 25.1 ± 1.0 and BDI score of 28.8 ± 1.7. They also had high levels of trait anxiety with a mean (±SEM) 62.1 ± 1.8 and of state anxiety 56.8 ± 2.9. Following therapy, patients showed a 50% reduction (or more) in clinical symptoms (mean ± SEM): HAM-D 6.6 ± 1.2 (Figure 1) and BDI 8.9 ± 1.4 and demonstrated moderate to high anxiety symptoms (mean ± SEM): trait anxiety 44.9 ± 3.1 and state anxiety 38.2 ± 2.5.

### Oxytocin

**Figure 2:**
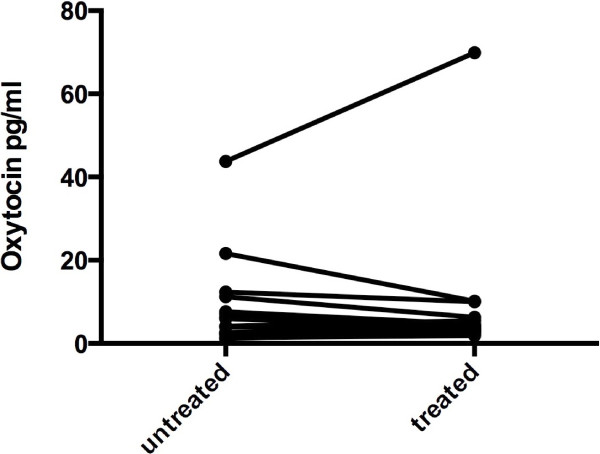
Arterial oxytocin concentrations in patients with MDD untreated and following SSRI treatment (p > .05).

There was no change in the plasma concentrations of oxytocin following treatment with an SSRI (Figure 2). There occurred no difference in the response between males and females.

### Cortisol

The mean (±SEM) plasma concentrations of cortisol (ng/ml) did not change significantly before (88.3 ± 6.8) to following (84.3 ± 6.2) treatment with an SSRI (p > .05). There occurred no difference in the response between males and females.

### Oxytocin, cortisol and psychopathology

There were no significant correlations between oxytocin and cortisol, or each of oxytocin, and cortisol with symptoms of MDD (p > .05).

## Discussion

Our findings indicate that oxytocin concentrations in patients with MDD are not influenced by effective treatment with an SSRI. While this outcome is in agreement with previous research that has demonstrated no change in serum oxytocin concentrations following various antidepressant treatments (pharmacological and ECT), in a sample that included both bipolar disorder and MDD [25], it is contrary to some preclinical data. For example, previous studies in rats showed that antidepressant (SSRI) medication can increase concentrations of oxytocin (e.g., [35]). Oxytocin mRNA expression in the PVN and SON has been shown to increase following stimulation via a variety of serotonin agonists (e.g., 1A, IB, 2A and 2C) [36]. In addition, both acute and chronic (2 week) administration of intraperitoneal citalopram or zimeldine (different antidepressants within the SSRI class) resulted in increased plasma oxytocin secretion [35]. In a recent study, a yellow fluorescent protein, *Venus*, was placed under control of the regulatory region of the gene encoding the oxytocin receptor in order to determine the expression pattern of oxytocin receptors throughout the brain. Widespread expression of oxytocin receptor containing cells were observed in these mice, in particular, a large co-localisation of Venus with tryptophan hydroxylase-positive neurons in the raphe nuclei [28]. Furthermore, intra-raphe infusion of oxytocin increased serotonin release within the median raphe, which was inhibited by serotonin 2A/C receptor antagonists. This lead the authors to speculate that the increased serotonergic activity induced by oxytocin may underlie its anxiolytic effects [28] and given the use of SSRI’s in MDD, these findings may also relate to their antidepressant-like effects [27]. We have, however, previously shown a reduction in serotonin turnover following SSRI therapy [32].

In our study, SSRIs were effective in treating symptoms of MDD in these patients (e.g., at least 50% reduction in symptoms following 12 weeks therapy). Although it is accepted that the HPA axis is commonly dysregulated in patients with MDD [12,13] the mechanisms are not well understood and the direction of dysregulation can furthermore vary. Nevertheless, sustained elevated activity of the HPA axis following antidepressant therapy has been previously linked to treatment resistance in patients with MDD (e.g., [37,38]). Hence, *a priori* selecting patients with a history of MDD that is resistant to treatment (with a matched healthy control group at baseline) may help to determine how dysregulation of the HPA axis (including oxytocin) relates to the pathophysiology of symptoms experienced by certain patients, and whether the HPA axis may represent a clinically relevant treatment target.

We did not show a change in the concentrations of cortisol in patients with MDD in response to treatment with SSRIs, suggesting that SSRIs did not significantly impact activity of the HPA axis in order to resolve symptoms. While measurements of cortisol and other stress hormones are notoriously difficult to control as the time of day, season, exercise, smoking, general health and the immediate events right before sampling can all affect biological measurement of cortisol [39], we controlled for time of day and season, caffeine, alcohol and tobacco smoking intake within 12 hours of study visits, and general health (as described previously, [32]). A limitation of the current study, however, is that hormones were not sampled across the day, on each of the study visits. Individuals with depression (compared with healthy controls) often demonstrate abnormal diurnal rhythm of cortisol with lower morning cortisol levels and higher evening levels [40]. Nevertheless, The fact that our results were in a small number of patients, and no differences in cortisol concentrations were detected from before relative to following treatment, attests to the robustness of our sampling procedure. Meta-analyses suggest, however, that MDD is associated with hypercortisolism at certain times of the day [40,41], hence, future research efforts should sample cortisol concentrations across the day.

In the current study, oxytocin concentrations in patients with MDD ranged between 2 pg/mL and 69 pg/mL with an overall mean of 8.52 pg/mL (untreated) and 8.72 pg/mL (treated). These findings are in agreement with previous research [25]. Several other studies have reported oxytocin concentrations that are significantly higher than those of the current study, however, the majority of these reports stem from observations in healthy populations. For example, in a large cohort of women and men (n = 323), oxytocin concentrations were averaged at 375.78 pg/mL [42]. Nevertheless, these concentrations ranged from 51.4 pg/mL to 2752.3 pg/mL, which suggests that in the general population, oxytocin concentrations are variable. Hence, future research should include comparison between patients and healthy control groups.

## Conclusions

In summary, this open label study presents data on arterial oxytocin and cortisol concentrations in patients with MDD before and following SSRI treatment. This has extended research in several ways. We have shown that oxytocin and cortisol concentrations in patients with MDD were not influenced by a single class of antidepressant treatment. This suggests that stress physiology is unlikely to contribute in a key manner to symptoms of MDD, or their resolution, for this group of patients who experience effective relief of their symptoms following SSRI therapy. It is well known that elevated activity of the HPA axis can impair a response to antidepressant treatment (including SSRIs). Thus, to better understand the contribution of HPA axis dysregulatoin to MDD, *a priori* selection of patients with a history of MDD that is resistant to treatment, may demonstrate potentially clinically important relationships between these physiological and psychological variables.

## Competing interest

The laboratory of Professor Lambert currently receives research funding from Medtronic, Abbott Pharmaceuticals, Servier Australia and Allergan. Professor Lambert has acted as a consultant for Medtronic and has received honoraria or travel support for presentations from Pfizer, Wyeth Pharmaceuticals, Servier and Medtronic. Dr Keating reports her patents (granted) in psychopharmacology. There are no financial competing interests associated with these.

## Authors’ contributions

The trial was conceived and designed by Dr B, Professor L, Dr D, Dr K and Professor T. Dr K interpreted the data and wrote the original draft of the manuscript. All authors read and approved the final manuscript.

## Pre-publication history

The pre-publication history for this paper can be accessed here:

http://www.biomedcentral.com/1471-244X/13/124/prepub
